# Metabolic Adaptation to Nutritional Stress in Human Colorectal Cancer

**DOI:** 10.1038/srep38415

**Published:** 2016-12-07

**Authors:** Masaaki Miyo, Masamitsu Konno, Naohiro Nishida, Toshinori Sueda, Kozo Noguchi, Hidetoshi Matsui, Hugh Colvin, Koichi Kawamoto, Jun Koseki, Naotsugu Haraguchi, Junichi Nishimura, Taishi Hata, Noriko Gotoh, Fumio Matsuda, Taroh Satoh, Tsunekazu Mizushima, Hiroshi Shimizu, Yuichiro Doki, Masaki Mori, Hideshi Ishii

**Affiliations:** 1 Department of Gastroenterological Surgery Osaka University Graduate School of Medicine, Suita, 565-0871, Japan; 2 Department of Frontier Science for Cancer and Chemotherapy Osaka University Graduate School of Medicine, Suita, 565-0871, Japan; 3 Faculty of Mathematics Kyushu University, Fukuoka, 819-0395, Japan; 4 Department of Cancer Profiling Discovery, Osaka University Graduate School of Medicine, Suita, 565-0871, Japan; 5 Cancer Research Institute of Kanazawa University, Kanazawa, Ishikawa, 920-1192, Japan; 6 Graduate School of Information Science and Technology, Osaka University, Suita, Osaka, 565-0871.

## Abstract

Tumor cells respond to their microenvironment, which can include hypoxia and malnutrition, and adapt their metabolism to survive and grow. Some oncogenes are associated with cancer metabolism via regulation of the related enzymes or transporters. However, the importance of metabolism and precise metabolic effects of oncogenes in colorectal cancer remain unclear. We found that colorectal cancer cells survived under the condition of glucose depletion, and their resistance to such conditions depended on genomic alterations rather than on *KRAS* mutation alone. Metabolomic analysis demonstrated that those cells maintained tricarboxylic acid cycle activity and ATP production under such conditions. Furthermore, we identified pivotal roles of GLUD1 and SLC25A13 in nutritional stress. GLUD1 and SLC25A13 were associated with tumor aggressiveness and poorer prognosis of colorectal cancer. In conclusion, GLUD1 and SLC25A13 may serve as new targets in treating refractory colorectal cancer which survive in malnutritional microenvironments.

Colorectal cancer is the third most frequently diagnosed cancer, fourth leading cause of cancer deaths worldwide, and accounts for 1.3 million new cases and 690,000 deaths annually^1^. Although effective treatment has led to improvements in survival, colorectal cancer remains a major global health problem^2^,^3^. Thus, it is necessary to develop additional novel and efficient treatments.

The physiology of tumor tissues differs from that of normal tissues in many aspects, the majority of which result from differences between the vasculatures of the two tissues^4^. Poorly formed tumor vasculature leads to a hypoxic microenvironment in which the nutrient levels are low and levels of waste products are high^5^. Tumor cells respond to such conditions and adapt their metabolism to survive and grow. Cancer cells are well known for having high rates of glucose consumption and lactate production despite bioavailability of sufficient oxygen for complete oxidation of glucose. This phenomenon is termed the Warburg effect^6^. Since the discovery of the Warburg effect, many researchers have studied the metabolism of cancer cells and tumor tissues. In cancer cells, glutamine, one of the most important nutrients as well as glucose, is reportedly metabolized more abundantly than other non-essential amino acids^7^. Glutamine metabolism not only provides a source for synthesis of macromolecules, such as lipids, proteins, and nucleotides, but also supports nicotinamide adenine dinucleotide phosphate (NADPH) production and anaplerosis in proliferating tumor cells^8^. This difference in metabolism between cancer and normal cells is expected to provide opportunities for development of innovative cancer treatments.

Several reports have indicated that tumor cells show changes in metabolism induced by oncogenes. *MYC* and *HIF-1* have been reported to regulate genes associated with glucose metabolism, such as the glucose transporter, *GLUT1*, hexokinase 2, pyruvate kinase M2 (*PKM2*), lactate dehydrogenase A, and pyruvate dehydrogenase kinase 1^9^. *MYC* is also postulated to stimulate glutamine metabolism via regulation of amino acid transporters (e.g., SLC1A5) and glutaminase. Moreover, expressions of the malic enzyme 1 (*ME1*) gene and malic enzyme 2 gene are inhibited by *p53*^10^. *KRAS* (G12D mutation) has an important role in regulating pancreatic tumor metabolism via stimulation of glucose uptake and activation of the hexosamine biosynthesis and pentose phosphate pathways^11^. Furthermore, there is a non-canonical pathway of glutamine in pancreatic ductal adenocarcinoma cells that is regulated by the *KRAS* oncogene^12^. However, the importance of glutamine metabolism and precise metabolic effects of oncogenes in colorectal cancer cells remain unknown.

The aim of this study is to elucidate metabolic adaptation to nutritional stress and the role of the involved oncogenes in human colorectal cancer. The present study showed that the metabolism of colorectal cancer, distinct from that of pancreatic cancer, depended on genomic alterations, which previously have been uncharacterized and was not restricted to *KRAS* mutation alone. Colorectal cancer can survive under the condition of glucose depletion while retaining TCA cycle activity. The cells’ survival relies on a delicate balance between energy and reactive oxygen species (ROS) production. Glutamate dehydrogenase 1 (GLUD1) and SLC25A13 have pivotal roles under glucose-deprived conditions and are associated with tumor aggressiveness and colorectal cancer prognosis.

## Results

### Survival of colorectal cancer cells under condition of glucose depletion

**Figure 1:**
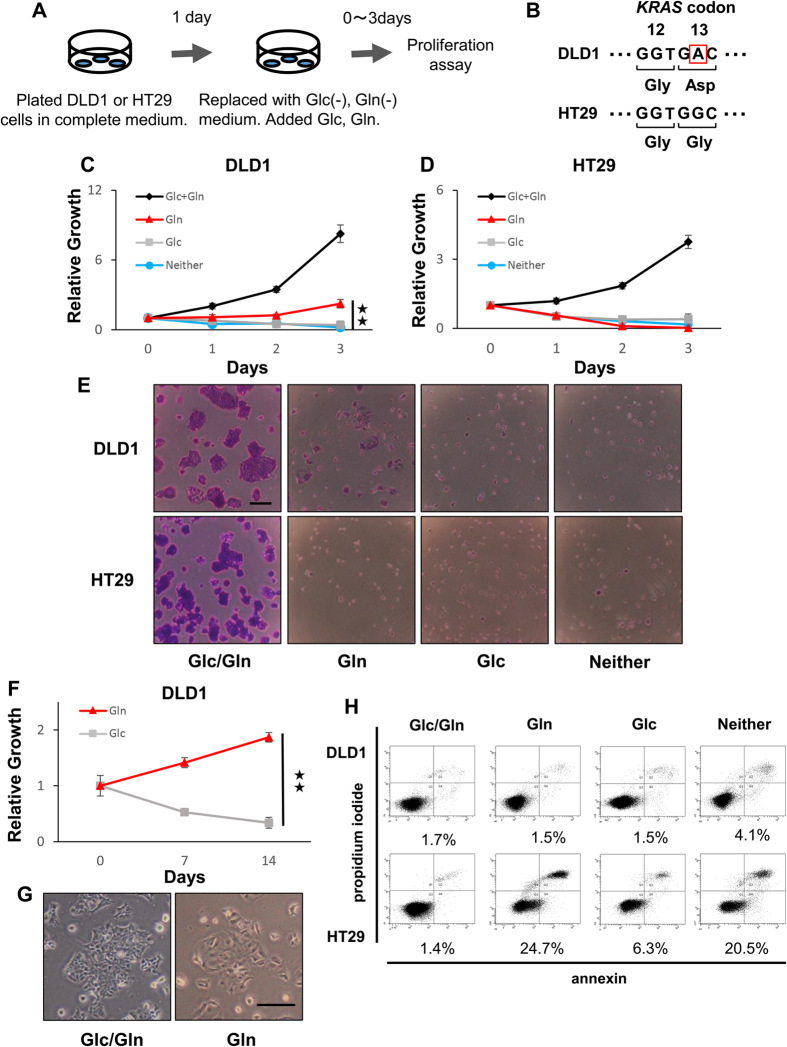
Colorectal cancer cell lines survive under glucose depleted conditions. (**A**) Cells were cultured in complete medium, which was replaced the following day with glucose- and glutamine-deficient media supplemented with 10% dialyzed fetal bovine serum and glucose (10 mM) or glutamine (2 mM), respectively. At the indicated time points, the cells were fixed in 80% methanol and stained with 0.1% crystal violet. OD determined the relative cell proliferation at 595 nm. Glc: glucose, Gln: glutamine. (**B**) RNA was extracted from DLD1 and HT29 cells, and the *KRAS* gene was PCR-amplified and sequenced. (**C,D**) Relative growth of DLD1 (**C**) and HT29 (**D**) under the indicated conditions. The difference in relative growth between Gln and Neither in DLD1 was significant. (**E**) The representative images are shown for day 3 (scale bar, 200 mm). (**F**) DLD1 cells survived for 2 weeks under the condition of glucose depletion. (**G**) The representative images are shown for day 14. (**H**) DLD1 and HT29 cells were cultured under the indicated conditions for 24 h. Apoptotic cells were analyzed by FACS using annexin V-FITC and propidium iodide. Representative results are shown. Data are presented as the mean ± SD of at least three independent experiments **P < 0.01).

Glucose and glutamine are two of the most abundant nutrients in plasma, and together, they account for most of the carbon and nitrogen metabolism occurring in mammalian cells. Both nutrients are essential for growth of pancreatic ductal adenocarcinoma cells with *KRAS* mutation^12^. To assess the role of glucose and glutamine in colorectal cancer cells, a proliferation assay was performed under various media conditions (Fig. 1A and Supplementary Fig. S1A). For the assay, we confirmed that DLD1 and HCT116 cells had a *KRAS* mutation at codon 13 involving a nucleotide change from GGC to GAC, and that HT29 and CaR1 cells did not have this *KRAS* mutation (Fig. 1B and Supplementary Fig. S1B). Notably, DLD1, HCT116, and CaR1 cells could survive under the glucose-deprived conditions (Fig. 1C–E and Supplementary Fig. S1C–E). Furthermore, DLD1 cells that had strong resistance to the condition of glucose depletion were able to survive for 14 days (Fig. 1F and G), and the passage of DLD1 cells was possible under that condition. The rate of apoptotic cells under the glucose-deprived conditions was lower in DLD1 cells than in HT29 cells (1.5% vs. 24.7%, respectively) (Fig. 1H). These findings show that colorectal cancer cells can survive under conditions of glucose depletion (glutamine sufficiency), which is profoundly different from pancreatic cancer cells in which both nutrients are indispensable.

### Resistance to glucose-deprived conditions in colorectal cancer depends on genomic alterations not restricted to *KRAS* mutation alone

**Figure 2:**
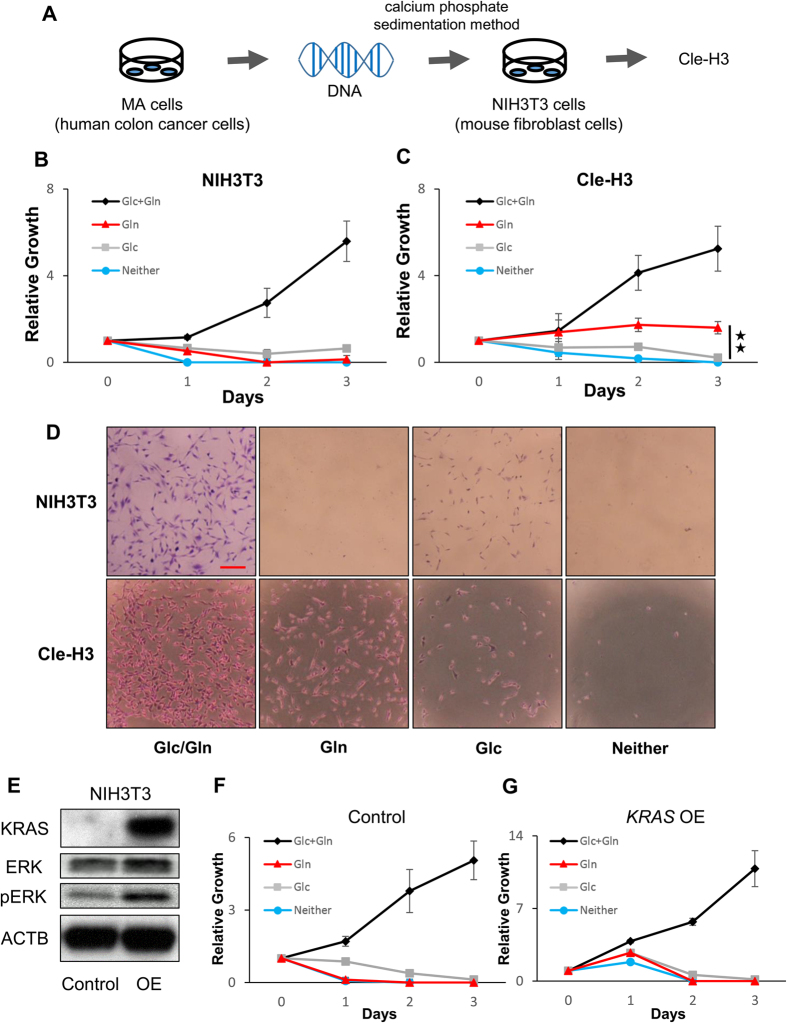
Resistance to glucose-deprived conditions in colorectal cancer depended on whole genomic alterations rather than on *KRAS* mutation alone. (**A**) The scheme shows the transfection methods of whole genomic DNA derived from a human colon cancer cell line (MA) harboring a *KRAS* mutation at codon 12 involving a nucleotide change from GGT to GAT. Transfection into NIH3T3 cells was performed using the calcium phosphate sedimentation method to establish Cle-H3 cells. (**B**,**C**) Relative growth of NIH3T3 (**B**) and Cle-H3 (**C**) under the indicated conditions. The difference in relative growth between Gln and Neither in Cle-H3 was significant. (**D**) Representative images are shown for day 3 (scale bar, 200 mm). (**E**) Western blot analysis of NIH3T3 cells transfected with control or *KRAS* overexpression (OE) vector. (**F**,**G**) Relative growth of control (**F**) and *KRAS*-overexpressing NIH3T3 cells (**G**) under the indicated conditions. Data are presented as the mean ± SD for at least three independent experiments (**P < 0.01).

Many reports indicate that the oncogene *KRAS* has an important role not only in cellular transformation and metabolic reprograming during tumorigenesis but also in chemotherapy for colorectal cancer, considering that mutant *KRAS* transduces oncogenic signaling and is the most reliable predictor for cancer response to anti-EGFR monoclonal antibodies^11^,^12^,^13^. We were interested in how genomic alterations, including alteration of the *KRAS* oncogene, in colorectal cancer influence cell proliferation under glucose-deprived conditions. To this end, mouse transformed NIH3T3 cells (Cle-H3) by human colorectal cancer genome with *KRAS* mutations were used in a proliferation assay^14^. The results showed that the Cle-H3 cells survived under glucose-deprived conditions, but the parental NIH3T3 did not (Fig. 2B–D), which suggested a role of the colorectal cancer genome in cell survival under glucose-deprived conditions. To assess whether these findings resulted solely from the oncogene *KRAS*, the proliferation assay using oncogenic *KRAS*-overexpressing NIH3T3 cells was similarly performed (Fig. 2E). The data showed that both *KRAS*-overexpressing NIH3T3 cells and control NIH3T3 cells did not survive under glucose-deprived conditions (Fig. 2F and G). Furthermore, knockdown of *KRAS* in the Cle-H3 and DLD1 cells did not influence their survival under glucose-depleted conditions (Supplementary Fig. S2A–F). These results demonstrated that resistance to the glucose-deprived conditions in colorectal cancer depended on uncharacterized genomic alterations that were not restricted to *KRAS* mutation alone.

### Metabolism of colorectal cancer was not restricted to *KRAS* mutation alone

**Figure 3:**
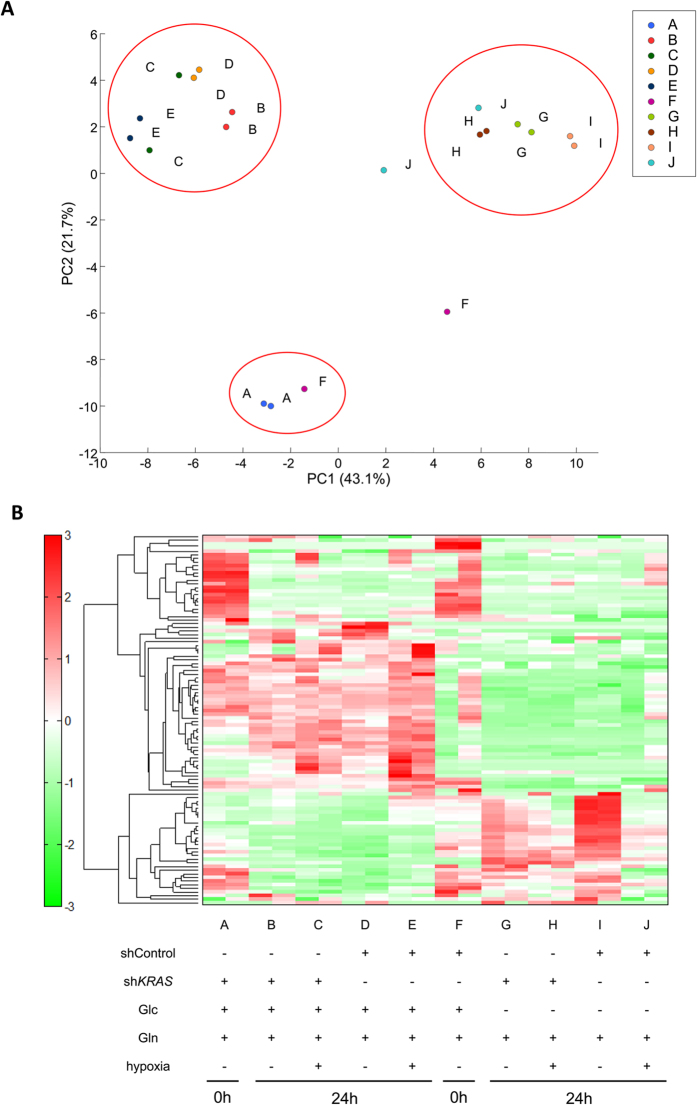
Metabolomic analysis for identification of metabolic effects of oncogene *KRAS*. (**A**,**B**) *KRAS* knockdown DLD1 cells and control cells were cultured at 3 × 10^6^ cells per 10-cm dish in 10 ml complete medium; the medium was replaced the following day with glucose(−) and glutamine(−) medium supplemented with 10% dialyzed fetal bovine serum. Glucose (10 mM) or glutamine (2 mM) was added, respectively. For hypoxia treatment, cells were incubated for 24 h with 1% O_2_. Principal component analysis (**A**) and hierarchical cluster analysis (**B**) performed under the indicated conditions at the indicated time points. The red circles indicate the groups that have similar principal component scores. Data for two independent experiments are presented.

We hypothesized that *KRAS* mutation alone may not markedly influence colorectal cancer metabolism. In the metabolomic analysis using control and *KRAS* knockdown DLD1 cells, which can survive under glucose-deprived conditions, we found that the metabolism of colorectal cancer did not depend on *KRAS* mutation alone but depended on the medium condition as well (Fig. 3A and B). In addition, k-means clustering and the Calinski criterion showed that k = 3 gave the optimal number of clusters, which indicated low dependence of metabolism of colorectal cancer on *KRAS* mutation alone (Supplementary Fig. S3A and B). Then, we measured the metabolites in plasma from wild type and *KRAS* mutation (Cre/LSL-KRASmut) mice. The principal component analysis showed no significant difference in cellular metabolites between two groups (Supplementary Fig. S3C). Considering that a mutation of another oncogene *BRAF* plays a role in tumor aggressiveness of retractable colorectal cancer in down stream pathway of KRAS, a role of BRAF was studied. We used two kinds of BRAF specific compounds, PLX4032 and PLX4720, in colorectal cancer HT29 cells in culture, and measured whole metabolites by CE-MS analysis. The data indicated that BRAF inhibition resulted in the suppression of metabolites in TCA cycle (Supplementary Fig. S4A–G), confirming that series of our experiments demonstrated the concept that mutant *KRAS* status does not predict glucose dependence in culture, and that glutamine fuels oxidative phosphorylation in TCA cycle, as observed in a stress condition such as BRAF specific inhibition in colorectal cancer cells. This observation suggests that the survival of colorectal cancer cells depends on a metabolomic mechanism different from those of other cancers, including pancreatic cancer^12^. On the basis of previous reports that showed that metabolism was important in DNA methylation^15^, DNA methylation analysis was performed under various conditions (Supplementary Fig. S3D). The data showed that DNA methylation frequency in colorectal cancer was not markedly influenced by depletion of glucose or glutamine.

### DLD1 cells retain TCA cycle activity under condition of glucose depletion

**Figure 4:**
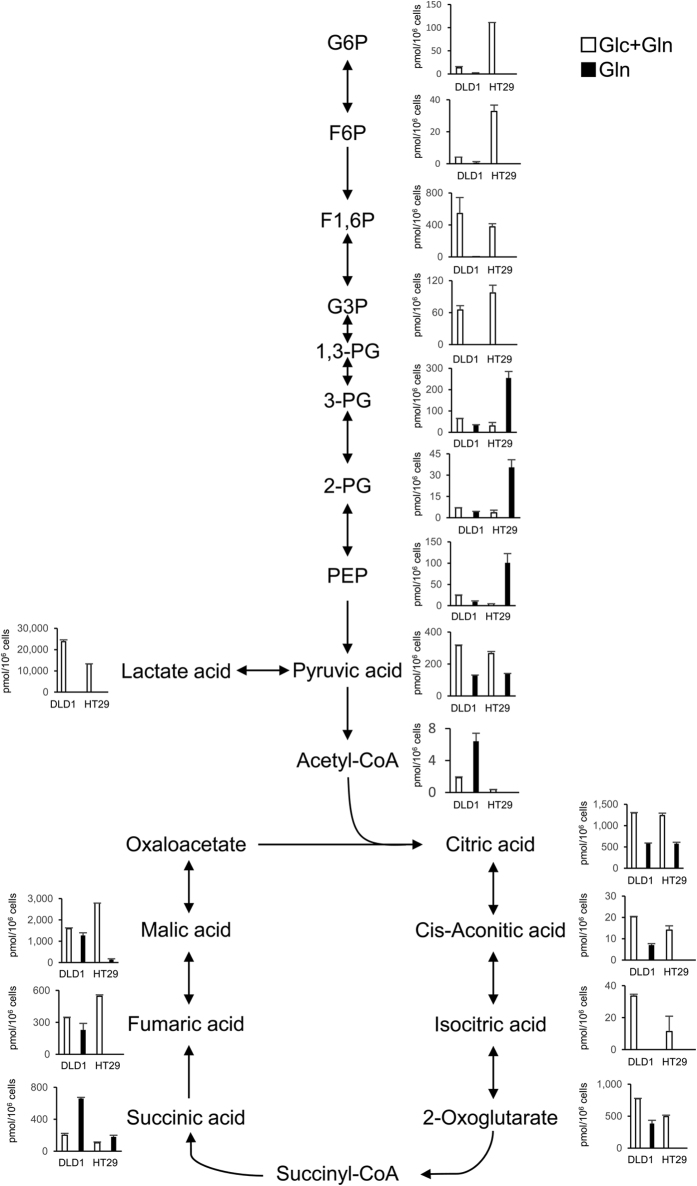
DLD1 cells retain TCA cycle activity under condition of glucose depletion. DLD1 and HT29 cells were cultured at 3 × 10^6^ cells per 10-cm dish in 10 ml complete medium; this medium was replaced the following day with glucose(−) and glutamine(−) medium supplemented with 10% dialyzed fetal bovine serum. Glucose (10 mM) or glutamine (2 mM) was added, respectively. Cells incubated for 24 h were analyzed. Data are presented as mean ± SD for two independent experiments.

To study the mechanism of resistance to glucose deprivation, the levels of glycolytic and TCA cycle metabolites were assessed by metabolomic analysis in living cells (DLD1 and HT29) pulse-chased for 24 h under glucose-deprived conditions. Notably, TCA cycle metabolites were maintained under the condition of glucose depletion in DLD1 cells but not in HT29 cells (Fig. 4). Under glucose-deprived conditions, the levels of glycerate 3-phosphate, 2-phosphoglycerate, and phosphoenolpyruvic acid increased in HT29 cells but not in DLD1 cells, which suggested that glucose depletion in HT29 cells induces gluconeogenesis. It is known that nicotinamide adenine dinucleotide consists of an oxidized form (NAD^+^) and a reduced form (NADH) and has a pivotal role in the electron transport chain in oxidative phosphorylation^16^; it is produced by glycolysis, the TCA cycle, and β-oxidation of fatty acids. The results showed that, in DLD1 cells, NADH decreased under glucose-deprived conditions compared with glucose- and glutamine-containing conditions, but NAD^+^ increased (Supplementary Fig. S5A). It is known that NADPH (reduced form) also has an oxidized form (NADP^+^) and provides reducing equivalents for various reactions related to biosynthesis; the molecule is used for generation of glutathione-scavenging ROS. The data showed that NADPH increased in DLD1 cells under glucose-deprived conditions, whereas NADPH decreased remarkably and NADP^+^ increased in HT29 cells (Supplementary Fig. S5B). Under glucose-deprived conditions, DLD1 and HT29 cells had few metabolites in the pentose phosphate pathway, which is the main source of nicotinamide adenine dinucleotide phosphate (Supplementary Fig. S5C), although other pathways, including those related to malic enzyme, isocitrate dehydrogenase, and nicotinamide nucleotide transhydrogenase, may be involved in its supply^17^. We performed the sphere formation assay, which is sensitive for intracellular redox status. Cells exhibiting high levels of ROS are susceptible to cell death and some cancer cells develop the redox pathway, which contributes to the cell survival and sphere formation. The results showed that the sphere formation frequency under the glucose depletion condition was greater in DLD1 cells when compared with HT29 cells, suggesting that cell survival was associated with the redox balance (Supplementary Fig. S5D). Together, these results indicate that DLD1 cells retain TCA cycle activity under the condition of glucose depletion and resist increases in ROS.

### Increase in amino acids levels in colorectal cancer cells under condition of glucose depletion

**Figure 5:**
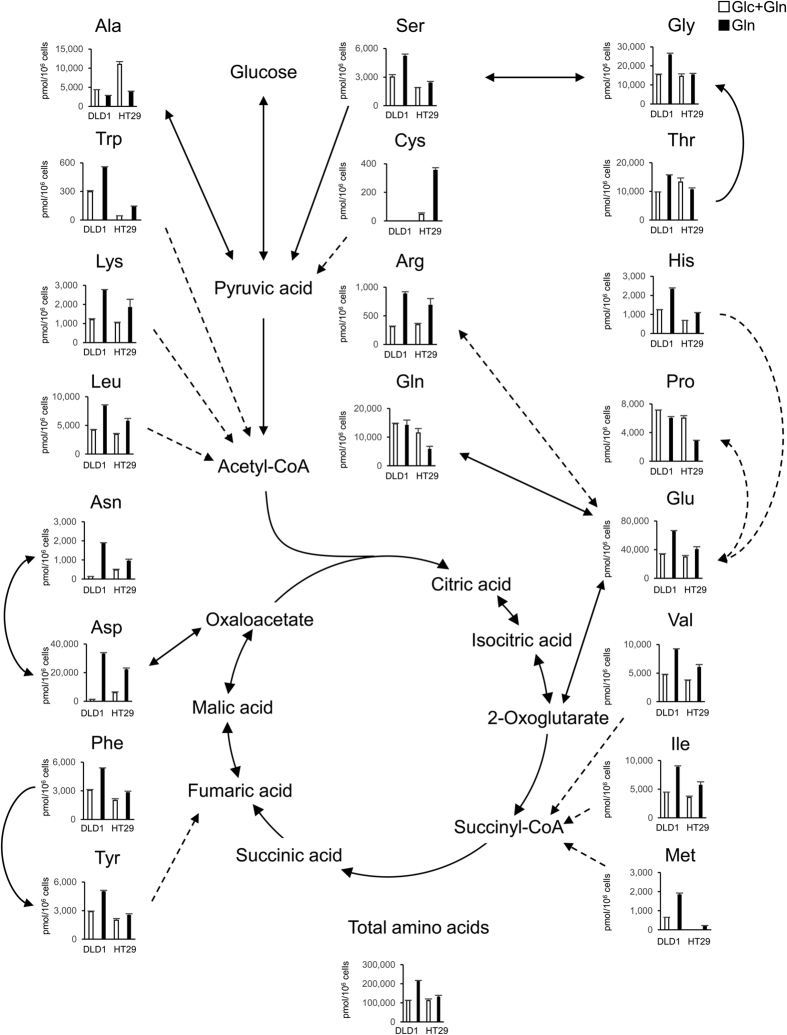
Increase in amino acids levels in colorectal cancer cells under condition of glucose depletion. DLD1 and HT29 cells were plated at 3 × 10^6^ cells per 10-cm dish in 10 ml complete medium; the medium was replaced the following day with glucose(−) and glutamine(−) media supplemented with 10% dialyzed fetal bovine serum. Glucose (10 mM) or glutamine (2 mM) was added, respectively. Cells incubated for 24 h were analyzed. Data are presented as mean ± SD for two independent experiments.

In addition to glycolytic and TCA cycle metabolites, the levels of amino acids in DLD1 and HT29 cells were studied by metabolomic analysis based on a method described in a previous report^18^ (Fig. 5). Compared with glucose- and glutamine-containing conditions, glucose-deprived conditions led to an increase in most of the amino acids in the DLD1 and HT29 cells. In particular, aspartic acid and asparagine levels increased remarkably in DLD1 cells (25.3 and 13.4 times, respectively). The total level of amino acids increased in both cell lines.

### Reliance of survival of DLD1 cells on the delicate balance between ROS and energy production

To adapt to nutritional stress, cells exposed to starvation have reportedly used autophagy to maintain ATP energy production and macromolecular synthesis^19^. Indeed, glucose depletion caused a decline in ATP levels in DLD1 and HT29 cells (Supplementary Fig. S6A) and induced autophagy in HT29 cells but not in DLD1 cells, as shown by LC3-II levels (Supplementary Fig. S6B). These findings suggest that the influence of autophagy on the increase in amino acids was limited to DLD1 cells (versus HT29 cells). Asparagine has reportedly been shown to suppress expression of C/EBP homologous protein (CHOP), which induces apoptosis^20^. In parallel with the effect of asparagine level, glucose depletion for 24 h induced CHOP in HT29 cells but not in DLD1 cells (Supplementary Fig. S6B), which suggested that the resistance to glucose-deprived conditions in colorectal cancer was partially associated with the level of asparagine. Given that the NADPH level in HT29 cells was low under glucose-deprived conditions, we studied ROS levels. The data showed that the level of ROS in HT29 cells under glucose-deprived conditions was 5.3 times higher than the levels under glucose- and glutamine-containing conditions, but it was only 1.7 times higher in DLD1 cells (Supplementary Fig. S6C). We were interested in whether the cell survival in glucose deprivation may depend on oxidative phosphorylation in mitochondria. Metformin reportedly inhibits the respiratory chain complex I associated with ATP generation in mitochondria^21^. As expected, the addition of metformin in culture under glucose-deprived conditions resulted in inhibition of growth of DLD1 cells in a concentration-dependent manner (Supplementary Fig. S6D). Taken together, the data support the idea that retention of resistance to glucose-deprived conditions may rely on oxidative phosphorylation and ROS level control.

### Significant association of *GLUD1* with resistance to glucose-deprived conditions in colorectal cancer

Glutaminolysis has a profound influence on the maintenance of redox balance and energy production. Under glucose-deprived conditions, its importance appears to further increase, considering that it may change according to the surrounding environment. To verify this hypothesis, gene microarray analysis was performed (Supplementary Fig. S7A) to identify candidate enzymatic genes related to the metabolism of glutamate and glutamine. *GLUD1* expression was greatly increased in DLD1 but not in HT29 cells. The results are consistent with those of a previous report in which GLUD1 was shown to have an important role under the condition of glucose deprivation in glioblastoma cells^22^. The changes in *GLUD1* expression were confirmed by quantitative real-time PCR (Supplementary Fig. S7B). Changes in glutamate dehydrogenase activity related to expression of *GLUD1* were also confirmed (Supplementary Fig. S7C). Knockdown of *GLUD1* in DLD1 cells under glucose-deprived conditions significantly decreased their growth (Supplementary Fig. S7D and E). Taken together, the present study showed that *GLUD1* was important in resistance to glucose-deprived conditions in colorectal cancer.

### Combined expression of GLUD1 and SLC25A13 is significantly associated with prognosis in colorectal cancer

**Figure 6:**
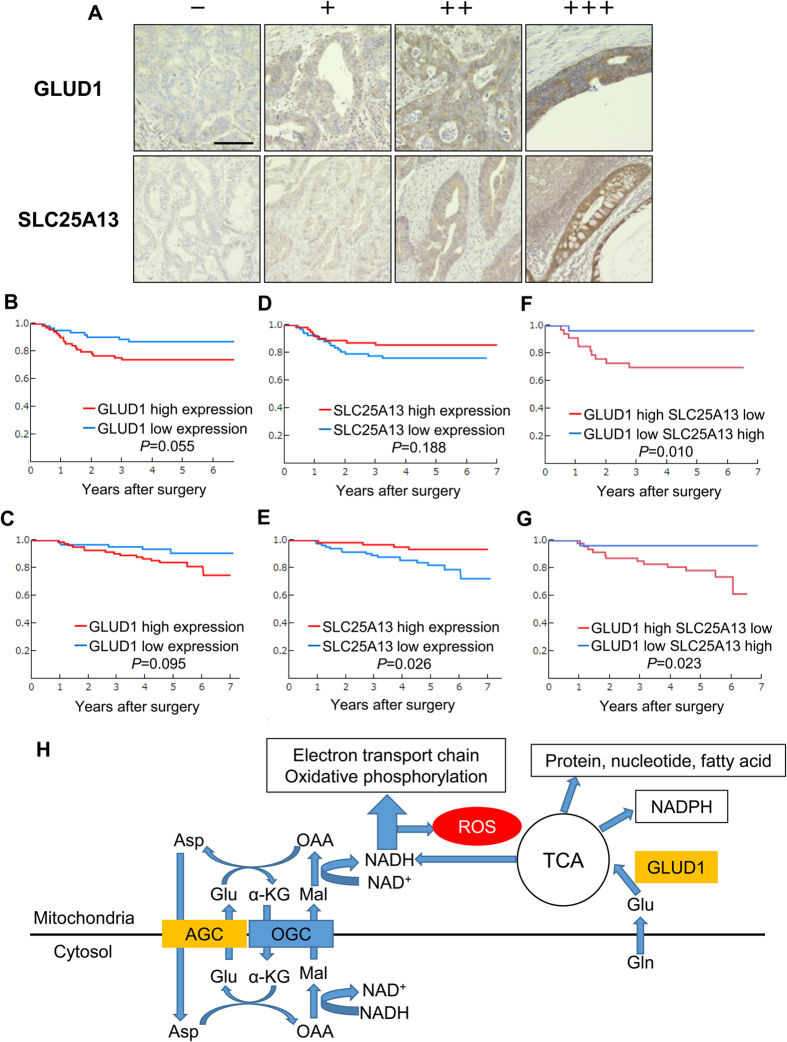
Combined expression of GLUD1 and SLC25A13 was shown to be significantly associated with prognosis in colorectal cancer. (**A**) Negative staining (−), positive staining (+), moderate staining (++), and strong staining (+++) for GLUD1 and SLC25A13 in colorectal cancer (scale bar, 200 mm). (B and C) Kaplan–Meier curves for recurrence-free survival (RFS) (**B**) and overall survival (OS) (**C**) according to GLUD1 expression. Differences between the two groups were evaluated by the log-rank test. Ordinate survival rate, abscissa years after surgery. (**D**,**E**) Kaplan–Meier curves for RFS (**D**) and OS (**E**) according to SLC25A13 expression. (F and G) Kaplan–Meier curves for RFS (**F**) and OS (**G**) according to combined expression of GLUD1 and SLC25A13. (**H**) Schematic overview of GLUD1-mediated Glu metabolism and SLC25A13-mediated ATP production. The citrin (SLC25A13) and aralar (SLC25A12; not shown) are components of aspartate-glutamate carrier (AGC), which functions collaboratively with oxoglutarate carrier (OGC) in the malate aspartate shuttle (MAS), a major intracellular pathway to transfer reducing equivalents. Glu: glutamic acid, Gln: glutamine, OAA: oxaloacetic acid, Asp: aspartic acid, α-KG: α-ketoglutaric acid, Mal: malic acid.

Cancer cells that have resistance to nutritional stress are expected to contribute to worse prognosis. Therefore, the published GSE17536 database was used for screening of genes related to the prognosis of colorectal cancer patients, as described previously^23^,^24^ (Supplementary Table S1). SLC25A13 was identified by analyses of the *GLUD1* gene and other genes involved in amino acid metabolism in all possible combinations as pairs. *SLC25A13* codes for mitochondrial aspartate–glutamate carrier (AGC), which has a central role in the malate–aspartate shuttle^25^. The reducing equivalents of NADH plus H^+^ through glycolysis are transferred from the cytosol to the mitochondria for the electron transport chain in oxidative phosphorylation by this shuttle, which is concerned with ROS and ATP production. In the DLD1 cells, the mRNA level of *SLC25A13* decreased under glucose-deprived conditions relative to the levels in glucose- and glutamine-containing conditions but increased conversely in HT29 cells (Supplementary Fig. S7F). Then we measured the enzyme activity of TCA related enzymes (succinate dehydrogenase and fumarase). The data showed that DLD1 had higher activity of succinate dehydrogenase, fumarase and malate dehydrogenase than HT29 cells did (Supplementary Fig. S8A–C). These findings suggest that the resistance to glucose-deprived conditions in colorectal cancer may result from inhibition of ROS production via repressed AGC. Subsequently, the relevance of GLUD1 and SLC25A13 expression in colorectal cancer with respect to clinicopathological characteristics and prognosis was evaluated by immunohistochemistry (Fig. 6A). High GLUD1 expression or low SLC25A13 expression was found to be associated with tumor aggressiveness, including depth of tumor invasion, lymph node and distant metastasis, lymphatic and venous invasion, and stage (Supplementary Table S2). Notably, high GLUD1 expression combined with low SLC25A13 expression had a higher association with tumor aggressiveness than did individual expression of each (Table 1). This combination also mirrored the effect on prognosis (Fig. 6B–G). There was no significant association between the individual expression of GLUD1 and SLC25A13. Then we assess the association of GLUD1 and PKM2 protein expression; the PKM2 is a splicing form of pyruvate kinase gene. The experimental data indicated that the GLUD1 expression was independent of PKM2 expression (Supplementary Fig. S8D), supporting the notion that both glucose metabolism and glutamine metabolism are important for the colorectal cancer. Taken together, colorectal cancer cells that can adapt to nutritional stress via regulation of GLUD1 and SLC25A13 contribute to tumor aggressiveness and result in worse prognosis (Fig. 6H).

## Discussion

We found that the metabolism of colorectal cancer cells that could survive under glucose-deprived conditions was significantly different from that of pancreatic ductal adenocarcinoma cells. In addition, the metabolism depended on genomic alterations that are uncharacterized and not restricted to *KRAS* mutation alone. In pancreatic ductal adenocarcinoma cells, a non-canonical glutamine pathway mediated by the oncogene *KRAS* that regulates glutamic-oxaloacetic transaminase 1 (*GOT1*) and *GLUD1* has been described^12^. In non-small cell lung cancer, *KRAS* has a profound influence on glutamine metabolism through regulation of *ME1* and *GOT1*, the high expression of which has been shown to correlate with poorer prognosis after radiotherapy^26^. A human breast carcinoma cell line that expresses an activated KRAS protein has been shown to have high glycolytic flux, low TCA cycle activity, and increased usage of glutamine for anabolic synthesis^13^. The solitary effects of oncogenic *KRAS* in metabolism are reportedly significant in various cancers; however, these effects were not observed for colorectal cancer in the present study. It would appear that in different cancers, metabolism varies, and the influence of oncogenes on metabolism is also diverse. Understanding the inherent metabolisms of different cancer types and how they are affected by oncogenes may lead to the development of specific cancer therapies.

In glutaminolysis, glutamine is first converted by glutaminase to glutamate and ammonia and then into α-ketoglutaric acid by either GLUD1 or less prominently by other transaminases. Jin *et al*. reported that expression of *GLUD1* was elevated in breast and lung cancer tissue relative to that in normal tissue^27^. In addition, the mammalian target of rapamycin complex 1 the activity of which is dysregulated in many cancers, activates GLUD1 via inhibition of *SIRT4*^28^. Cancer cells enhance glutaminolysis via positive regulation of *GLUD1* to respond to increased demand of glutamine, which is used as a carbon source for energy generation, a component of protein and nucleotide, and in production of glutathione and NADPH. On the basis of the results of immunohistochemistry for 104 colorectal cancer specimens, Liu *et al*. reported that GLUD1 expression was high and associated with poorer prognosis^29^. Our results showing an association between GLUD1 expression and prognosis in colorectal cancer were consistent with those of Liu *et al*. Enhanced glutaminolysis by increased expression and activity of GLUD1 under nutritional stress may contribute to these findings. A recent study investigated regulation of redox homeostasis by GLUD1. Fumarate, the level of which is controlled by GLUD1, has been shown to positively regulate a ROS-scavenging enzyme, glutathione peroxidase 1^27^. Our results showed that the increase in ROS levels under glucose-deprived condition was suppressed in DLD1 cells in which expression and activity of GLUD1 increased. Our results support those of the study by Jin *et al*. (Figs S5C, S6B and S6C).

A mutation in *SLC25A13* coding for citrin has been shown to cause citrin deficiency that results in type-II citrullinemia and is characterized by hyperammonemia, steatohepatitis, and neuropsychiatric symptoms^25^. Although expression of *SLC25A13* is known to be high in the liver, the significance of its expression in the colon has not been understood fully. In the present study, SLC25A13 expression was observed in the glandular cells of normal colorectal tissue and colorectal cancer tissue by immunohistochemistry (Fig. 6A). Mutations of *SLC25A13* reportedly lead to hepatic overload and increase the risk of hepatocellular carcinoma^30^. Shohet *et al*. reported that higher expression of *SLC25A13* correlated with a poorer outcome in neuroblastoma^31^. However, there are only a few studies that have examined the relevance of *SLC25A13* to cancer. To the best of our knowledge, this is the first study to identify the importance of *SLC25A13* in colorectal cancer. We showed that SLC25A13 was negatively associated with the depth of tumor invasion, extent of lymph node metastasis, distant metastasis, lymphatic invasion, and stage in colorectal cancer. Furthermore, SLC25A13 was profoundly correlated with colorectal cancer prognosis (Fig. 6D–G and Table S2). In addition, colorectal cancer cells were found to adapt to nutritional stress through regulation of *SLC25A13*. Therefore, *SLC25A13* may have an important role in development of colorectal cancer.

In conclusion, we found that the metabolism of colorectal cancer, which is different from that of pancreatic cancer, depended on genomic alterations that are uncharacterized and not restricted to *KRAS* mutation alone. Colorectal cancer cells that have resistance to glucose depletion retain TCA cycle activity and rely on the delicate balance between ROS production and ATP production. GLUD1 and SLC25A13 have pivotal roles in nutritional stress and are associated with tumor aggressiveness and poorer prognosis of colorectal cancer. These proteins may serve as new targets in the treatment of refractory colorectal cancer.

## Materials and Methods

### Cell lines, culture and serum

The human colorectal cancer cell line HT29 was obtained from the ATCC (Manassas, VA, USA). Other colorectal cancer cell lines included DLD1, which was obtained from the Cell Resource Center for Biomedical Research Institute of Development, Aging, and Cancer (Tohoku University), and HCT116 and CaR1, which were obtained from the Japan Cancer Research Resources Bank (JCRB, Tokyo, Japan). Mouse embryonic fibroblast cell lines, parental NIH3T3, and transformed NIH3T3 cells by whole genomic DNA derived from a human colon cancer cell line (MA) were purchased from the RIKEN Cell Bank (Tsukuba, Japan). Cells were cultured in Dulbecco’s modified Eagle’s medium (DMEM D6046; Sigma Aldrich, St. Louis, MO, USA) containing 10% fetal bovine serum (FBS), 100 U/ml penicillin, and 100 μg/ml streptomycin (Life Technologies, Carlsbad, CA, USA) at 37 °C in a humidified incubator with 5% CO_2_. Mice serum were obtained under anesthesia from wild type and *KRAS* mutation (Cre/LSL-KRASmut) BL6 mice, which were purchased from the Jackson laboratory (Bar Harbor, ME, USA), and maintained under the ethical agreement of animal facility at Osaka University (24–122; approved by chairman professor Kaneda).

### Cell proliferation assay

Cells were added to 24-well plates at 10,000–15,000 cells per well in 0.5 ml of medium that was replaced the following day with glucose- and glutamine-deficient medium (DMEM D5030; Sigma Aldrich) supplemented with 10% dialyzed FBS (#26400–044; Invitrogen, Carlsbad, CA). Glucose (Wako Pure Chemical Industries, Osaka, Japan) and glutamine (Nacalai Tesque, Kyoto, Japan) were added. After cells were fixed in 80% methanol and stained with 0.2% crystal violet, the relative cell proliferation was quantified by absorbance at 595 nm.

### Apoptosis detection

Apoptotic cells were analyzed using an annexin V [fluorescein isothiocyanate (FITC)-conjugated] apoptosis kit (K101–400; BioVision, Mountain View, CA, USA). In brief, cells growing on 6-cm dishes at 1–2 × 10^6^ cells/dish for 24 h were loaded with 0.5 ml binding buffer, 5 μl annexin V-FITC, and 5 μl propidium iodide. After incubation at room temperature for 5 min in the dark, annexin V-FITC binding and PI staining were analyzed by flow cytometry.

### Transfection of vector

Cells overexpressing *KRAS* were generated using a pCMV6-Entry plasmid containing the *KRAS*^G12V^ sequence with SgfI–MluI restriction sites (OriGene, Rockville, MD, USA) and Fugene6 (Roche Applied Science, Indianapolis, USA). *KRAS* knockdown cells were generated using the Lenti-vpak Packaging Kit (OriGene). A lentiviral shRNA construct, NM_033360 that targets human *KRAS* was obtained from the MISSION TRC-Hs1.0 library (Sigma). Control cells were transfected using the same procedure but with an empty control vector. DLD1 cells were selected using 2 μg/ml puromycin to establish stable cell lines.

### Western blot analysis

Western blot analysis was performed as previously described^32^. KRAS antibody (SAB1404011; Sigma Aldrich; 1:250 dilution), ERK (#9102; Cell Signaling Technology, Beverly, MA; 1:1000 dilution), phosphorylated ERK (#9101 S; Cell Signaling Technology; 1:1000 dilution), CHOP (#5554; Cell Signaling Technology; 1:1000 dilution), Bcl-2 (#2870; Cell Signaling Technology; 1:1000 dilution), and ACTB antibody (A2066; Sigma Aldrich; 1:1000 dilution) were used.

### Metabolomic analysis

Cells were cultured at 3 × 10^6^ cells per 10-cm dish in 10 ml complete medium; the medium was replaced the following day with glucose(−) and glutamine(−) medium supplemented with 10% dialyzed fetal bovine serum. Glucose (10 mM) or glutamine (2 mM) was added, respectively. For hypoxia treatment, cells were incubated for 24 h with 1% O_2_. After culture medium was aspirated from the dish, the cells were washed twice using 5% mannitol solution and treated with 800 μl of methanol and 550 μl of Milli-Q water containing internal standards [H3304–1002; Human Metabolome Technologies (HMT), Tsuruoka, Japan]. The metabolite extracts were centrifuged at 2,300 g at 4 °C for 5 min. Next, to remove macromolecules, 800 μl of the upper aqueous layer was filtered by centrifugation using a Millipore 5-kDa cutoff filter at 9,100 g at 4 °C for 120 min, and resuspended in 50 μl of Milli-Q water for metabolomic analysis. Metabolomic analysis was performed using a C-SCOPE package in HMT and capillary electrophoresis–time-of-flight mass spectrometry for cation analysis and capillary electrophoresis–tandem mass spectrometry for anion analysis^33^,^34^. To obtain information regarding the peak, including the m/z ratio, migration time (MT), and peak area, the peaks were identified using automatic integration software (MasterHands; Keio University, Tsuruoka, Japan and MassHunter Quantitative Analysis B.04.00; Agilent Technologies, Santa Clara, CA, respectively). Peak areas were normalized against those of the internal standards, and the relative area values were further normalized by sample amounts. Hierarchical cluster analysis and principal component analysis were performed using HMT’s proprietary softwares, PeakStat and SampleStat, respectively.

### Immunohistochemical analysis

Immunohistochemical analysis was performed as previously described^32^. Colorectal cancer tissue samples were obtained from 151 consecutive patients who underwent surgery at the Osaka University Hospital between 2006 and 2009. None of the patients had received chemotherapy or radiotherapy before surgery. All patients provided their written informed consent for use of their clinical samples in this study, which was approved by the Institutional Review Board. GLUD1 antibody (ab166618; Abcam; 1:500 dilution), SLC25A13 antibody (PA5–21991; Thermo Fisher Scientific; 1:500 dilution) were used.

### Evaluation of staining

According to a method described in a previous report^35^, we scored the samples as follows: (A) fraction of positive stained cells: ≤5% scored 0, 6%–25% scored 1, 26%–50% scored 2, 51%–75% scored 3, and ≥75% scored 4; (B) intensity of staining: no staining scored 0, weak staining scored 1, moderate staining scored 2, and strong staining scored 3. The immunoreactivity score (IRS) was obtained by multiplying both parameters. The samples were divided into two groups as follows: low expression (IRS < 6) and high expression (IRS ≥ 6) groups.

### Statistical analysis

Data were indicated as the mean ± standard deviation (SD). We performed Student’s t-test and Fisher’s exact test to determine statistically significant differences using JMP Pro 10 software (SAS Institute, Cary, NC, USA). The Kaplan–Meier method was used to assess recurrence-free survival and overall survival. A P-value < 0.05 was considered to indicate statistical significance.

## Additional Information

**How to cite this article**: Miyo, M. *et al*. Metabolic Adaptation to Nutritional Stress in Human Colorectal Cancer. *Sci. Rep.* **6**, 38415; doi: 10.1038/srep38415 (2016).

**Publisher’s note:** Springer Nature remains neutral with regard to jurisdictional claims in published maps and institutional affiliations.

## Supplementary Material

Supplementary Information

## Figures and Tables

**Table 1:** Statistical results of immunohistochemical analysis for GLUD1 and SLC25A13 in colorectal cancer.

Clinicopathological factors	Classification	N	GLUD1 high SLC25A13 low	GLUD1 low SLC25A13 high	*P*
Patient background					
Sex	Male	46	28	18	NS^a^
	Female	31	21	10	
Age	<65	40	28	12	NS
	≥65	37	21	16	
Tumor characteristics					
Histological type^b^	tub1, tub2, pap	72	47	25	NS
	por, muc	5	2	3	
Depth of tumor invasion	Tis, T1, T2	25	6	19	<0.001
	T3, T4	52	43	9	
Lymph node metastasis	Positive	45	37	8	<0.001
	Negative	32	12	20	
Distant metastasis	Positive	15	14	1	0.008
	Negative	62	35	27	
Lymphatic invasion	Positive	59	45	14	<0.001
	Negative	18	4	14	
Venous invasion	Positive	22	19	3	0.009
	Negative	55	30	25	
Stage	0, I, II	28	8	20	<0.001
	III, IV	49	41	8	

^a^NS, not significant.

^b^Tub1, well-differentiated adenocarcinoma; tub2, moderately differentiated adenocarcinoma; pap, papillary adenocarcinoma; por, poorly differentiated adenocarcinoma; muc, mucinous carcinoma.
