# Supplementary material for: Metabolic Adaptation to Nutritional Stress in Human Colorectal Cancer
Source: Sci Rep. 2016 Dec 7;6:38415. doi: 10.1038/srep38415 (PMC5141444; doi:10.1038/srep38415)

Supplementary information

[Title]

Metabolic Adaptation to Nutritional Stress in Human Colorectal Cancer

[Authors]

Masaaki Miyo[1], Masamitsu Konno[2], Naohiro Nishida[2], Toshinori Sueda[1], Kozo Noguchi[1], Hidetoshi Matsui[5], Hugh Colvin[1,3], Koichi Kawamoto[1,2], Jun Koseki[3], Naotsugu Haraguchi[1], Junichi Nishimura[1], Taishi Hata[1], Noriko Gotoh[6], Fumio Matsuda[4], Taroh Satoh[2], Tsunekazu Mizushima[1], Hiroshi Shimizu[4], Yuichiro Doki[1,2,3], Masaki Mori[1,2,3], and Hideshi Ishii[2,3*]

Supplementary figure legends

Supplementary Figure S1. Colorectal cancer cell lines survived under conditions of glucose depletion. (A) Cells were plated in complete medium that was replaced the following day with glucose(−) and glutamine(−) medium supplemented with 10% dialyzed fetal bovine serum; glucose (10 mM) or glutamine (2 mM) was added, respectively. At the indicated time points, the cells were fixed in 80% methanol and stained with 0.1% crystal violet. OD determined the relative proliferation at 595 nm. Glc: glucose, Gln: glutamine. (B) RNA was extracted from HCT116 and CaR1 cells, and the *KRAS* gene was PCR-amplified and sequenced. (C and D) Relative growth of HCT116 (C) and CaR1 (D) under the indicated conditions. The

difference in relative growth between Gln and Neither in HCT116 and CaR1 is significant. (E) Representative images are shown for day 3 (scale bar, 200 mm). Data are presented as the mean ± SD of at least three independent experiments **P < 0.01).

Supplementary Figure S2. Knockdown of *KRAS* in Cle-H3 and DLD1 cells did not have a large effect on cell survival under conditions of glucose depletion. (A) Cle-H3 cells transfected with a control shRNA or shRNA to *KRAS* were analyzed for KRAS expression by Western blot analysis. (B and C) Relative growth of control (B) and *KRAS* knockdown Cle-H3 cells (C) under the indicated conditions. The difference in relative growth between Gln and Neither in the control and *KRAS* knockdown Cle-H3 cells is significant. (D) DLD1 cells transfected with a control shRNA or shRNA to *KRAS* were analyzed for KRAS expression by Western blot analysis. (E and F) Relative growth of control (E) and *KRAS* knockdown DLD1 cells (F) under the indicated conditions. The difference in relative growth between Gln and Neither in the control and *KRAS* knockdown DLD1 cells is significant. Data are presented as the mean ± SD for at least three independent experiments (**P < 0.01).

Supplementary Figure S3. DNA metabolomic and methylation analysis. (A) K-means clustering applied to *KRAS* knockdown DLD1 cells and control cells in the metabolomic

analysis. (B) Calinski criterion for *KRAS* knockdown DLD1 cells and control cells in the metabolomic analysis. k = 3 was selected as the optimal number of clusters. (C) In vivo assessment of metabolites in plasma of peripheral blood from wild type and *KRAS* mutant (Cre/LSL-KRASmut) mice. The data of principal component analysis are shown. (D) DLD1 cells plated at 3×10^6 cells per 10-cm dish in 10 ml of complete medium, which was replaced the following day with glucose(−) and glutamine(−) medium supplemented with 10% dialyzed fetal bovine serum; glucose (10 mM) or glutamine (2 mM), was added respectively. Cells incubated for 24 h were analyzed. The Spearman's rank correlation coefficients between Glc + Gln and Glc, Glc + Gln and Gln, and Glc and Gln are 0.989, 0.988, and 0.987, respectively.

Supplementary Figure S4. Metabolomic analysis for colon cancer using BRAF inhibitors. (A-G) The effect of *BRAF* mutation specific compounds, PLX4032 and PLX4720, in colorectal cancer HT29 cells with *BRAF* V600 mutation. The cells were cultured in the medium with compounds, and subjected to metabolomic analysis. Data are presented as the mean ± SD for three independent experiments.

Supplementary Figure S5. Metabolomic analysis for DLD1 and HT29 cells. (A) NAD^+ and NADH levels of DLD1 and HT29 cells under the indicated conditions. (B) $NADP^+$ and NADPH

levels of DLD1 and HT29 cells under the indicated conditions. (C) The metabolites associated with the pentose phosphate pathway of DLD1 and HT29 cells under the indicated conditions. Data are presented as the mean ± SD for two independent experiments. (D) Sphere forming assay of DLD1 and HT29 cells. These cells were cultured the medium including Glutamine and with or without glucose. Data are presented as the mean ± SD for eight independent experiments. ($^{*}P < 0.05$)

Supplementary Figure S6. Difference between DLD1 and HT29 cells under glucose-deprived conditions. (A) AMP, ADP, and ATP levels of DLD1 and HT29 cells under the indicated conditions from metabolomic analysis. Data are presented as the mean ± SD for two independent experiments. (B) DLD1 and HT29 cells plated in complete medium that was replaced the following day with glucose(−) and glutamine(−) medium supplemented with 10% dialyzed fetal bovine serum; glucose (10 mM) or glutamine (2 mM) was added, respectively. Cells incubated under the indicated conditions for 12 h or 24 h were analyzed by Western blot analysis. (C) DLD1 and HT29 cells were cultured under the indicated conditions for 24 h and their ROS levels were analyzed. (D) DLD1 cells were plated in complete medium, which was replaced the following day with glutamine medium only. The cells were incubated with the indicated concentration of metformin for 24 h. Cell numbers were determined from absorbance

at 595 nm (OD 595). Data are presented as the mean ± SD of at least three independent experiments (*P < 0.05, **P < 0.01, and N.S; not significant).

Supplementary Figure S7. Importance of GLUD1 and SLC25A13 under conditions of glucose depletion. (A) Gene microarray analysis indicates the change in expression of enzymatic genes related to glutaminolysis according to the medium conditions. Fold change is the expression of the gene under glucose-deprived conditions divided by that under glucose- and glutamine-containing conditions. Fold change ratio is the proportion to DLD1 fold change divided by the proportion of HT29 fold change. The highest 20 fold change ratios are shown. (B) The expression levels of *GLUD1* in DLD1 and HT29 determined by quantitative real-time PCR. (C) Glutamate dehydrogenase activity in DLD1 and HT29 cells under the indicated conditions. (D) The expressions of *GLUD1* in control and *GLUD1* knockdown DLD1 cells. (E) Knockdown of *GLUD1* in DLD1 cells significantly decreased cell growth under glucose-deprived conditions. The cell numbers were determined from absorbance at 595 nm (OD 595). (F) The expression levels of *SLC25A13* in DLD1 and HT29 determined by quantitative real-time PCR. Data are presented as the mean ± SD for at least three independent experiments (*P < 0.05, **P < 0.01).

Supplementary Figure S8. Study of TCA enzyme activity. The enzyme activities are measured in indicated cells. The results are shown in succinate dehydrogenase (A), fumarase (B) and malate dehydrogenase (C). The data are shown by the relative enzyme activity. (D) Assessment of GLUD1 and PKM2 by immunohistochemistry of colorectal cancer patients. Graph indicates the number of patients with negative (0), positive (1), moderate (2), and strong staining (3) in GLUD1 protein.

Experimental procedures

Reagents. LC-3 antibody (#2775; Cell Signaling Technology; 1:1000 dilution), CHOP (#5554; Cell Signaling Technology; 1:1000 dilution), and Bcl-2 (#2870; Cell Signaling Technology; 1:1000 dilution) were used for Western blot analysis and PKM2 antibody (#4053; Cell Signaling Technology; 1:500 dilution) were used for immunohistochemical analysis. A lentiviral shRNA construct NM_005271 targeting human *KRAS* was obtained from the MISSION TRC-Hs1.0 library (Sigma).

K-Means clustering. We applied k-means clustering to the data for some number of clusters, and then selected the appropriate number of clusters in the metabolomic analysis. Here we used the R package "Vegan" for implementing this strategy. To select the number of clusters we used the Calinski criterion of variance ratio criterion. Suppose we have a set of N observations with p genes. Then VRC for the result of the clustering with k clusters is given by

$$VRC_k = \frac{SS_B/(k-1)}{SS_W/(N-k)'}$$

where SS_B is the between-class variation and SS_W is the within-class variation, respectively, defined as

$$SS_B = \sum_{j=1}^{k} \frac{N_j}{N}(\bar{\boldsymbol{x}}_j - \bar{\boldsymbol{x}})^T(\bar{\boldsymbol{x}}_j - \bar{\boldsymbol{x}}), \quad SS_w = \frac{1}{N}\sum_{j=1}^{k}\sum_{i=1}^{N_j}(\boldsymbol{x}_{ij} - \bar{\boldsymbol{x}}_j)^T(\boldsymbol{x}_{ij} - \bar{\boldsymbol{x}}_j),$$

where N_j is the number of subjects for the jth cluster, $\boldsymbol{x}_{ij}$ is the p dimensional vector of the ith subject in the jth cluster, $\overline{\boldsymbol{x}}_j = \frac{1}{N_j}\sum_{i=1}^{N_j} x_{ij}$ is the sample mean for the jth cluster, $\overline{\boldsymbol{x}} = \frac{1}{N}\sum_{j=1}^{k}\sum_{i=1}^{N_j} x_{ij}$ is the overall sample mean, and T is the transpose of the vector. We calculated the value of VRC_k for $k = 2, \ldots, 10$ and then selected the value of k that minimized VRC_k as the optimal number of clusters.

DNA methylation analysis. DNA methylation analysis was performed in a manner described previously [1]. Briefly, genomic DNA was extracted from DLD1 cells under various media conditions using the QIAamp DNA Mini Kit (Qiagen) according to the manufacturer’s instructions. The DNA methylation analysis was performed using the Illumina infinium assay with the HumanMethylation450 BeadChip (Illumina, San Diego, CA, USA). The methylation levels of the samples were calculated as AVG-beta values, which are quantitative scores ranging from “0,” indicating completely unmethylated, to “1,” indicating completely methylated. The association between two samples was determined using Spearman rank correlation coefficients.

Enzyme activity assay. Enzyme activity assay, such as succinate dehydrogenase (Bio Vision, Milpitas, CA, USA), fumarase (Bio Vision), malate dehydrogenase (Bio Vision) and glutamate

dehydrogenase (Bio Vision) were performed according to the manufacturer's instructions using DLD1 and HT29 cell lines.

Quantitative real-time PCR analysis. Quantitative real-time PCR analysis was performed in a manner as previously described [2]. The following primers were used: GLUD1 primers (forward: 5′-GGGATTCTAACTACCACTTGCTCA-3′ and reverse: 5′-AACTCTGCCGTGGGTACAAT-3′) and SLC25A13 primers (forward: 5′-GAAAGTGCTACGCTATGAAGG-3′ and reverse: 5′-GCAGTCTATCACTCCGCTGT-3′). ACTB primers (forward: 5′-GATGAGATTGGCATGGCTTT-3′ and reverse: 5′-CACCTTCACCGTTCCAGTTT-3′) were used as a normalization control.

Detection of ROS. The ROS-Glo™ H2O2 Assay (Promega, Madison, WI, USA) was used to measure the level of ROS generation, according to the manufacturer's instructions.

Gene microarray analysis. A 3D-Gene Human Oligo chip 25k (Toray Industries Inc., Tokyo, Japan) was used (25,370 distinct genes). For efficient hybridization, this microarray has three-dimensions, with spaces between the probes and cylinder stems and 70-mer oligonucleotide probes on the top. Total RNA was labeled with Cy5 using the Amino Allyl MessageAMP II

RNA Amplification Kit (Applied Biosystems, CA, USA). The Cy5-labeled RNA pools were incubated in hybridization buffer for 16 h. The hybridization was performed using the supplier's protocols (www.3d-gene.com). Hybridization signals were scanned using a 3D-Gene Scanner (Toray Industries) and processed with 3D-Gene Extraction software (Toray Industries). The raw data for each spot was normalized by subtraction of the mean intensity of the background signal determined from all blank spot signal intensities, with 95% confidence intervals. Raw data intensities >2 standard deviations (SD) of the background signal intensity were considered to be valid. Detected signals for each gene were normalized using the global normalization method (the median of the detected signal intensity was adjusted to 25).

Screening for genes related to survival of patients with colorectal cancer. The published GSE17536 database (http://www.ncbi.nlm.nih.gov/geo/query/acc.cgi?acc=GSE17536) was used for screening of genes related to the prognosis of colorectal cancer patients, as described previously [3,4]. The database included the microarray data of 177 patients. The *GLUD1* gene and other genes involved in amino acid metabolism were divided into two groups (low- and high-expression groups) on the basis of the median point of expression. The relevance of the aforementioned gene expression levels to recurrence-free survival (RFS) and overall survival

(OS) was evaluated. Furthermore, the *GLUD1* gene and other genes involved in amino acid metabolism were analyzed in all possible combinations, as pairs.

References

1 Maekawa, R. *et al.* Genome-wide DNA methylation analysis reveals a potential mechanism for the pathogenesis and development of uterine leiomyomas. *PLoS One* **8**, e66632, doi:10.1371/journal.pone.0066632 (2013).

2 Miyo, M. *et al.* Tumour-suppressive function of SIRT4 in human colorectal cancer. *Br J Cancer* **113**, 492-499, doi:10.1038/bjc.2015.226 (2015).

3 Smith, J. J. *et al.* Experimentally derived metastasis gene expression profile predicts recurrence and death in patients with colon cancer. *Gastroenterology* **138**, 958-968, doi:10.1053/j.gastro.2009.11.005 (2010).

4 Koseki, J. *et al.* Mathematical analysis predicts imbalanced IDH1/2 expression associates with 2-HG-inactivating beta-oxygenation pathway in colorectal cancer. *Int J Oncol* **46**, 1181-1191, doi:10.3892/ijo.2015.2833 (2015).

Supplementary Table S1. *P* values of Kaplan–Meier curves determined by log-rank test based on combined expression of genes and *GLUD1* in colorectal cancer

	High vs. low[a]		High + *GLUD1* high vs. low + *GLUD1* low[b]		Low + *GLUD1* high vs. high + *GLUD1* low[c]	
	DFS[d]	OS[e]	DFS[d]	OS[e]	DFS[d]	OS[e]
BCAT1	0.00007902	0.01383914	0.0027241	0.0195556	0.0081476	0.4333889
SLC7A7	0.04413727	0.16265310	0.1068359	0.0771770	0.1942637	0.8001879
PLOD1	0.05890775	0.03413584	0.1278615	0.0224243	0.2724754	0.5375584
SMS	0.07581540	0.24721376	0.1397965	0.1060222	0.3103095	0.9183245
PYCR1	0.21732239	0.39282367	0.1585720	0.9170847	0.7801279	0.1887446
SLC7A2	0.14122641	0.15646871	0.1719559	0.0723272	0.3862529	0.7694548
GSS	0.02956344	0.14893891	0.1739596	0.9243782	0.0956376	0.1038742
SLC6A6	0.23120464	0.98625690	0.2077044	0.3896906	0.6003472	0.4217307
SDS	0.21533237	0.49668466	0.2130660	0.2241863	0.5978422	0.6920695
GCLM	0.16000421	0.91493731	0.2228660	0.3853191	0.4922143	0.4787854
SLC25A13	0.07095548	0.16604378	0.2367775	0.8487596	0.1098087	0.0876453
PTS	0.21583192	0.11846848	0.2375957	0.0433005	0.5813396	0.6236715
ASPA	0.06076742	0.22384804	0.3053650	0.9127834	0.1139112	0.0883234
CDO1	0.37801853	0.27038434	0.3159943	0.1121055	0.7504426	0.9448644
ALDH6A1	0.25325875	0.39480854	0.3237785	0.1840148	0.4956272	0.8594258
ASL	0.06002846	0.05869352	0.3322362	0.7188505	0.0728656	0.0488365
BCKDK	0.33353127	0.76223407	0.3441228	0.5204730	0.7356064	0.3391540
SLC3A1	0.13652423	0.09686447	0.3571656	0.6661429	0.1651694	0.0251309
PEPD	0.07794064	0.33924683	0.3744418	0.8022521	0.0770239	0.1756084
SLC25A15	0.08981591	0.01444905	0.3841746	0.4103593	0.0781661	0.0110836
DCT	0.10432850	0.94869062	0.3933236	0.4396401	0.1764711	0.4347466
GLDC	0.09308280	0.35100724	0.4237452	0.9294370	0.1687132	0.1622166
ADI1	0.09897649	0.23280885	0.4785068	0.9869977	0.0922207	0.0639336
PAH	0.16196355	0.32628557	0.4821713	0.9112175	0.1982828	0.1536502
DARS	0.67108713	0.91195135	0.4972288	0.3547549	0.8413761	0.4556322

HPD	0.62258465	0.23834061	0.5068417	0.8976036	0.9467148	0.1143760
HGD	0.64027333	0.16256710	0.5257442	0.0828969	0.9028107	0.9212853
FARS2	0.17028538	0.55757716	0.5335365	0.6524902	0.1224403	0.2683769
GAD2	0.81898368	0.80120823	0.5344070	0.3415462	0.8577689	0.5170983
GLS2	0.69936428	0.80576117	0.5361820	0.2890377	0.9797010	0.5887098
SLC6A14	0.91070283	0.77199031	0.5457749	0.6904409	0.6961958	0.2108337
TYR	0.83159661	0.56461578	0.5540039	0.8132663	0.8577977	0.1846971
BCKDHB	0.22206459	0.14146987	0.5542535	0.7833678	0.1511172	0.0489960
NFS1	0.66394277	0.11287062	0.5568169	0.7801590	0.9288860	0.0493450
ALDH18A1	0.17765626	0.54855935	0.5580024	0.2586497	0.1880818	0.7107278
MARS2	0.22157714	0.11504630	0.5699414	0.8646501	0.2276972	0.0498150
ATF4	0.96443769	0.21547701	0.5791437	0.0422321	0.6679836	0.8586675
GAD1	0.86773613	0.10941122	0.5903254	0.0498871	0.8864730	0.5698806
BAAT	0.23764059	0.04093854	0.6110777	0.4597271	0.2106261	0.0182749
DDO	0.35476613	0.11883911	0.6329630	0.7683910	0.3769468	0.0725573
GCLC	0.81394481	0.82119636	0.6449222	0.4440456	0.7869429	0.3916011
ALDH4A1	0.17715629	0.98867128	0.6502596	0.3848800	0.1649812	0.4966506
SLC7A4	0.33164478	0.36847871	0.6586576	0.9392727	0.2866153	0.1131211
SCLY	0.29268701	0.10866713	0.6665060	0.7645420	0.2949579	0.0722994
GGT1	0.31634329	0.16012184	0.6716089	0.8101703	0.2731208	0.0895297
SARS2	0.95436388	0.79494670	0.6853884	0.2897586	0.8171910	0.5960351
MTHFR	0.93890398	0.31523188	0.6922212	0.9293244	0.8048039	0.1283800
ASRGL1	0.30288969	0.89379143	0.6977812	0.5038397	0.2553650	0.3768239
FAH	0.97632094	0.43345952	0.7188968	0.6999071	0.6646058	0.1875080
SLC25A12	0.97217755	0.51264338	0.7271934	0.2078655	0.7441650	0.7829472
SLC7A5	0.92116033	0.89536674	0.7362802	0.2568205	0.9280920	0.6243818
GOT2	0.42195882	0.29147653	0.7587487	0.8722801	0.4485721	0.1433883
HDC	0.43321370	0.57853139	0.7620926	0.6615605	0.3881344	0.2396527
IDO1	0.88592961	0.11416682	0.7694657	0.9305158	0.6732063	0.0608290

GLUD2	0.98664143	0.21331615	0.7838426	0.2094708	0.2733217	0.7978968
YARS	0.84883466	0.57296619	0.7949519	0.1765380	0.6064238	0.7760312
ARG1	0.92285097	0.85367293	0.8076305	0.2686356	0.7062700	0.5720795
QDPR	0.75839514	0.36333626	0.8083657	0.1420793	0.5359836	0.9151179
MAT2B	0.91444383	0.86477255	0.8132003	0.5315140	0.6415946	0.2706072
AARS	0.94111257	0.59973300	0.8257304	0.0830492	0.7050019	0.7921848
GOT1	0.38591499	0.32644962	0.8287514	0.2011231	0.2479877	0.8964182
KARS	0.46864015	0.81235485	0.8397756	0.2751051	0.3801746	0.6224745
SLC7A8	0.81684049	0.03392578	0.8489772	0.4967303	0.6303884	0.0322893
DDAH1	0.42002043	0.16845704	0.8727886	0.8974371	0.2422626	0.0365850
MAT1A	0.47572189	0.27998776	0.8781198	0.9617432	0.4374138	0.1055677
MSRA	0.38261972	0.48833914	0.9014682	0.6784588	0.2816435	0.1585560
SLC7A9	0.57908402	0.62193759	0.9168687	0.1391939	0.4475640	0.7732097
SLC7A6	0.45847617	0.27327566	0.9318619	0.1387574	0.7850272	0.8915688
WARS	0.63676693	0.66988189	0.9480187	0.5201967	0.5589936	0.3147727
GCSH	0.59703353	0.93837841	0.9619964	0.3157090	0.5026426	0.5869020
GSTZ1	0.74399896	0.79325262	0.9651242	0.3248978	0.4946329	0.4985355
RARS	0.62888959	0.94853428	0.9710785	0.4431702	0.4204049	0.3978496
DDAH2	0.72061830	0.87463235	0.9878636	0.5258820	0.5453270	0.3912562
MCCC2	0.63517335	0.44915937	0.9912342	0.7534987	0.4516191	0.0913706
GLUD1	0.65509971	0.25483888				

[a] High expression of genes vs. low expression

[b] The genes high expression + *GLUD1* high expression vs. the genes low expression + *GLUD1* low expression

[c] The genes low expression + *GLUD1* high expression vs. the genes high expression + *GLUD1* low expression

[d] DFS, disease-free survival

[e] OS, overall survival

Yellow cells denote *P* < 0.11

Supplementary Table S2. Statistical results of immunohistochemical analysis for GLUD1 and SLC25A13 in colorectal cancer

Clinicopathological factors	Classification	N	GLUD1 high expression	GLUD1 low expression	*P*	SLC25A13 high expression	SLC25A13 low expression	*P*
Patient background								
Sex	Male	90	46	44	NS[a]	36	54	NS[a]
	Female	61	39	22		28	33	
Age	<65	75	42	33	NS	38	38	NS
	≥5	76	43	33		26	49	
Tumor characteristics								
Histological type[b]	tub1, tub2, pap	140	80	60	NS	58	82	NS
	por, muc	11	5	6		6	5	
Depth of tumor invasion	Tis, T1, T2	63	24	39	<0.001	37	26	<0.001
	T3, T4	88	61	27		27	61	
Lymph node metastasis	Positive	72	50	22	0.003	21	51	0.002
	Negative	79	35	44		43	36	
Distant metastasis	Positive	18	14	4	NS	1	17	<0.001
	Negative	133	71	62		63	70	
Lymphatic invasion	Positive	112	71	41	0.005	40	72	0.008
	Negative	39	14	25		24	15	
Venous invasion	Positive	41	30	11	0.015	14	27	NS
	Negative	110	55	55		50	60	
Stage	0, I, II	75	31	44	<0.001	43	32	<0.001
	III, IV	76	54	22		21	55	

[a] NS, not significant

[b] tub1, well-differentiated adenocarcinoma; tub2, moderately differentiated adenocarcinoma; pap, papillary adenocarcinoma; por, poorly differentiated adenocarcinoma; muc, mucinous carcinoma.

Supplementary Figure S1.

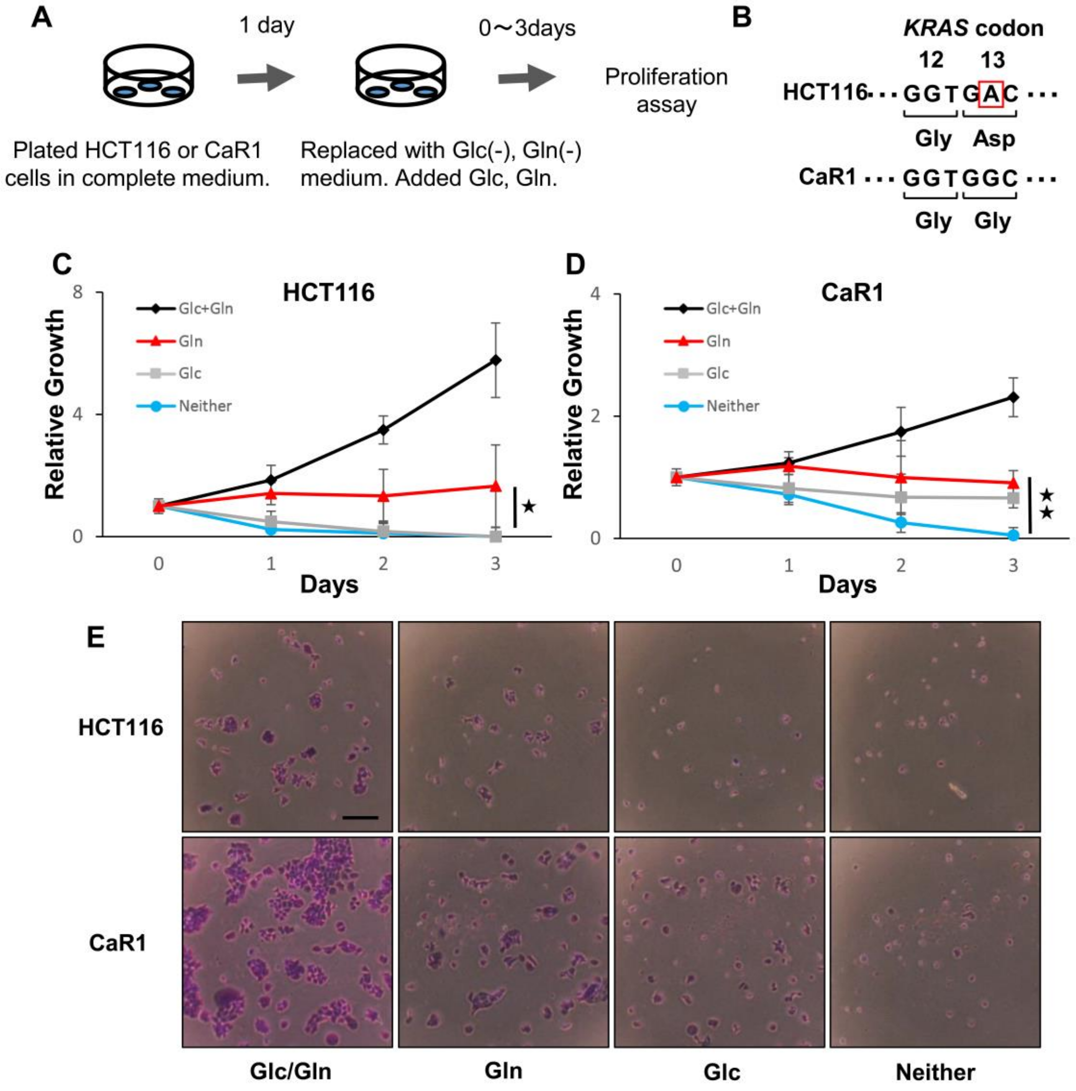

Supplementary Figure S2.

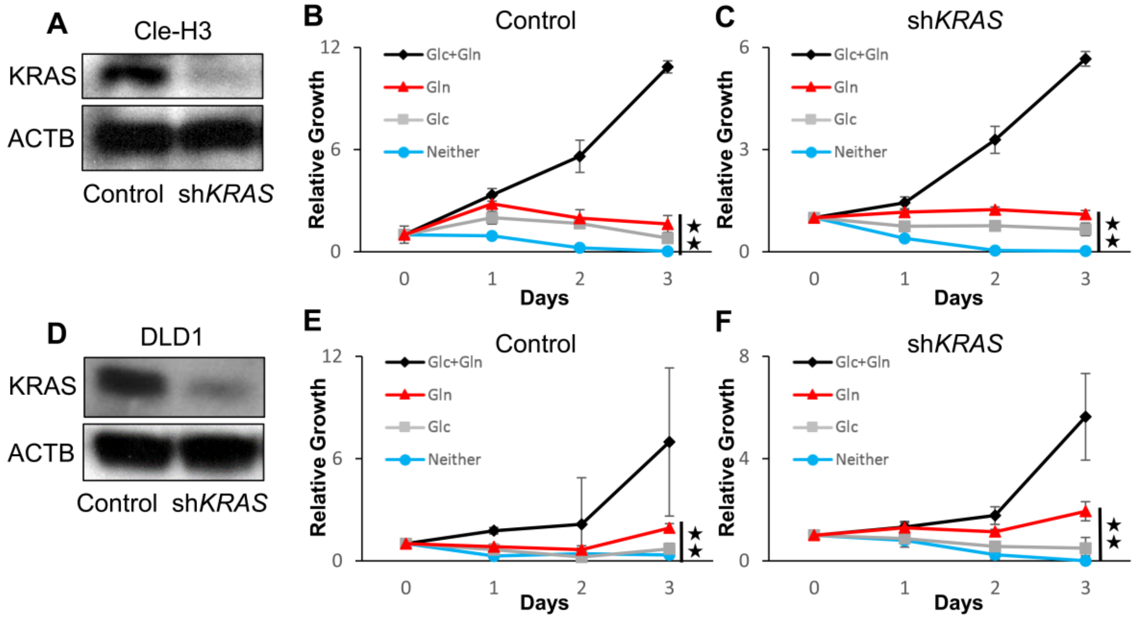

Supplementary Figure S3.

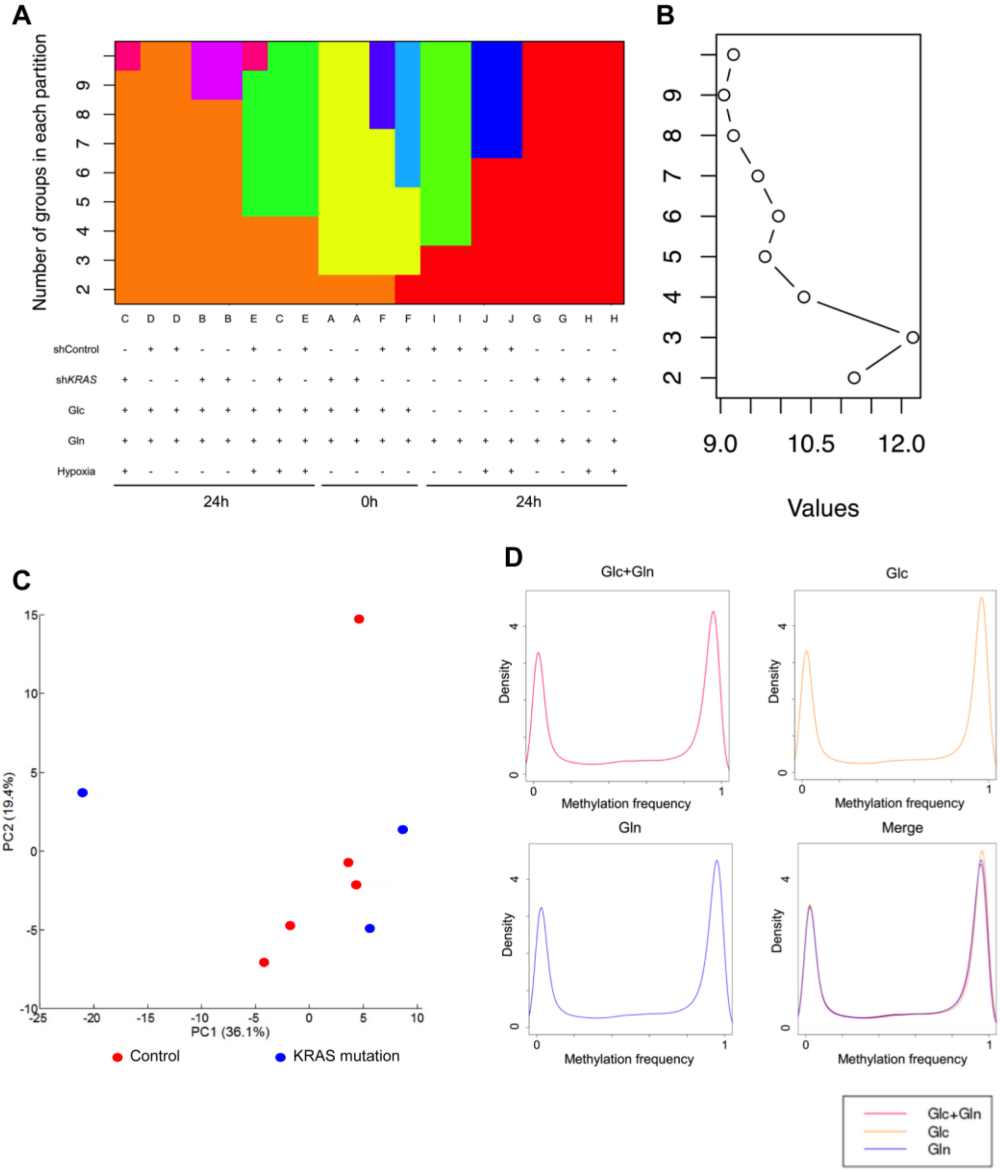

Supplementary Figure S4.

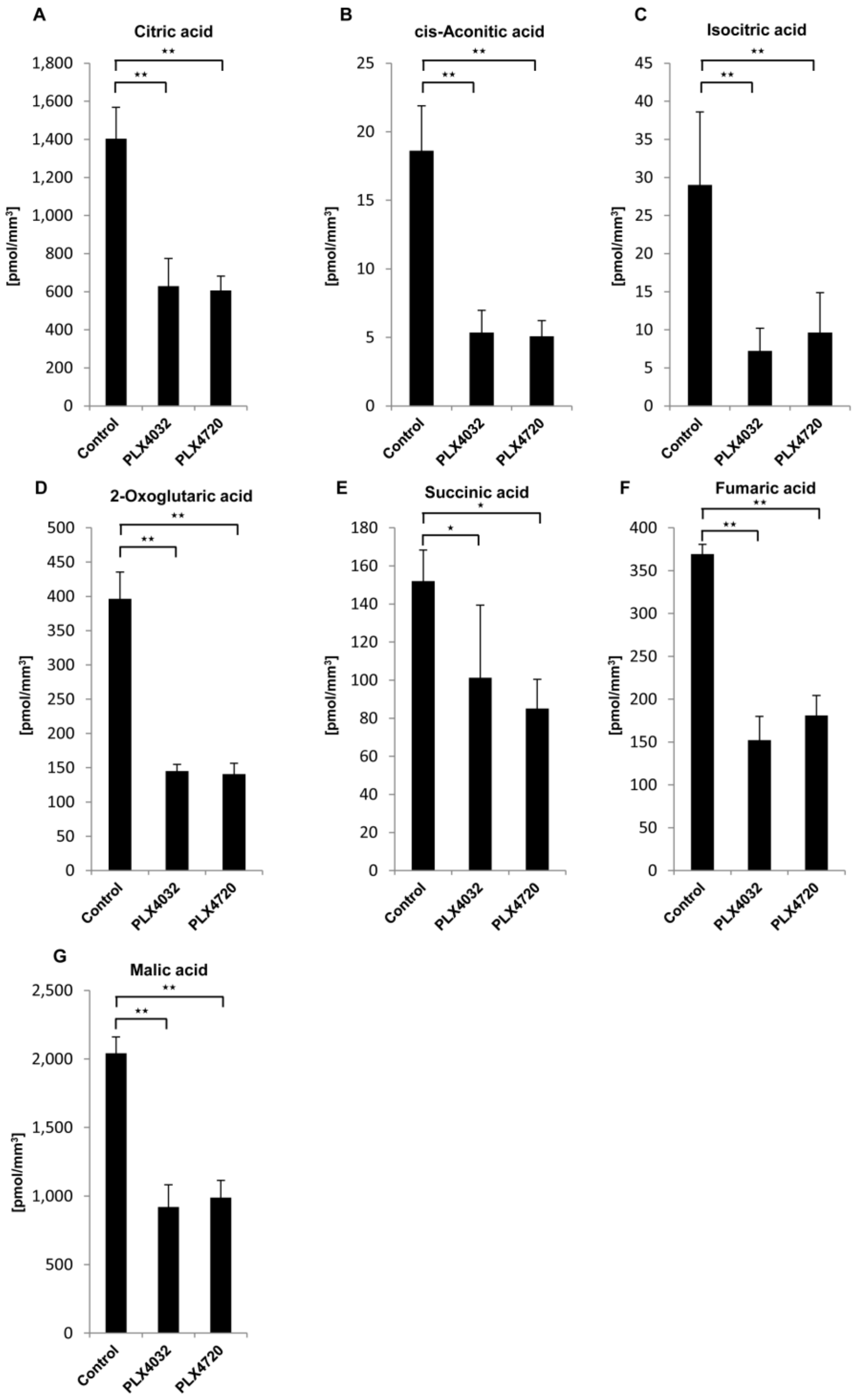

Supplementary Figure S5.

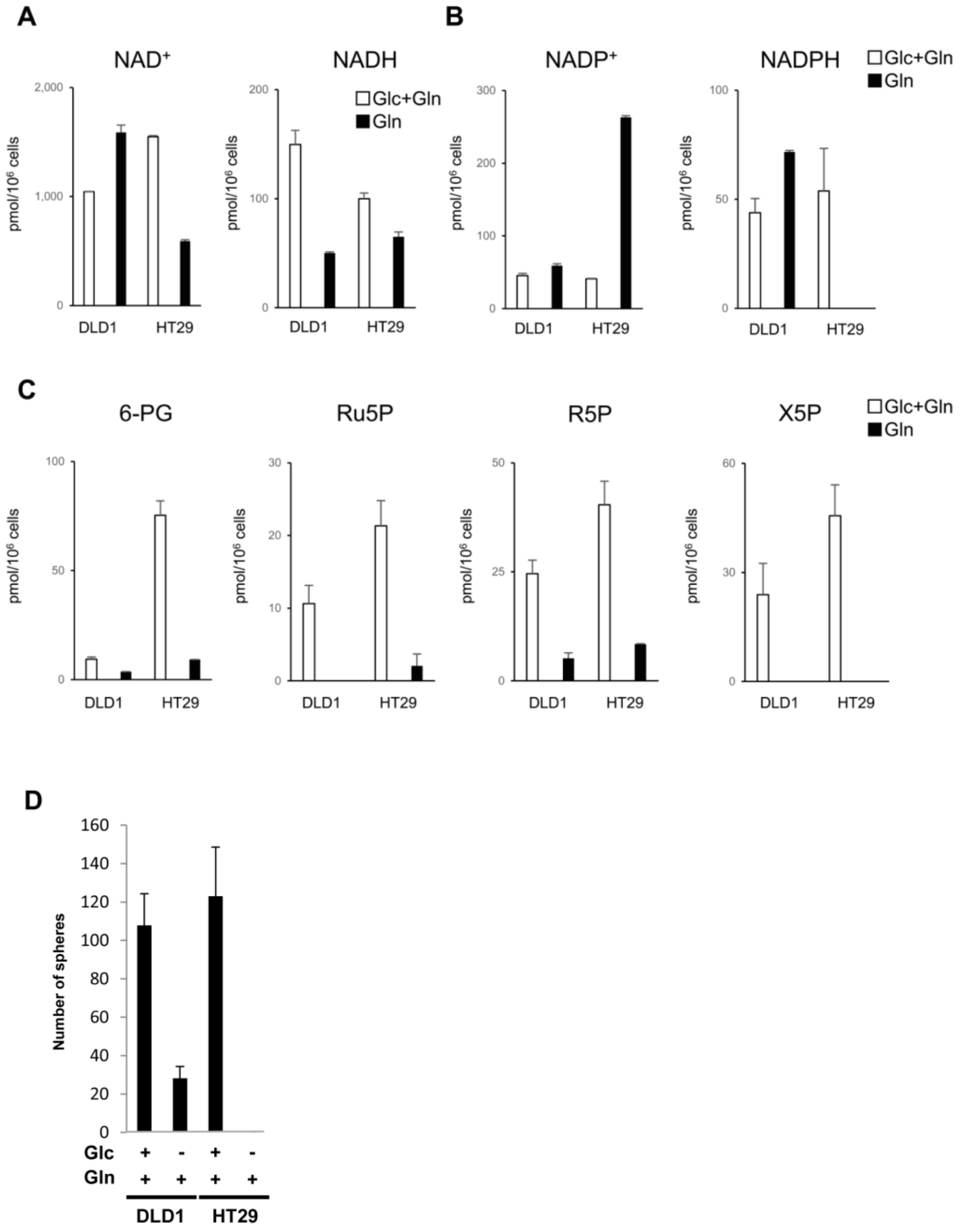

Supplementary Figure S6.

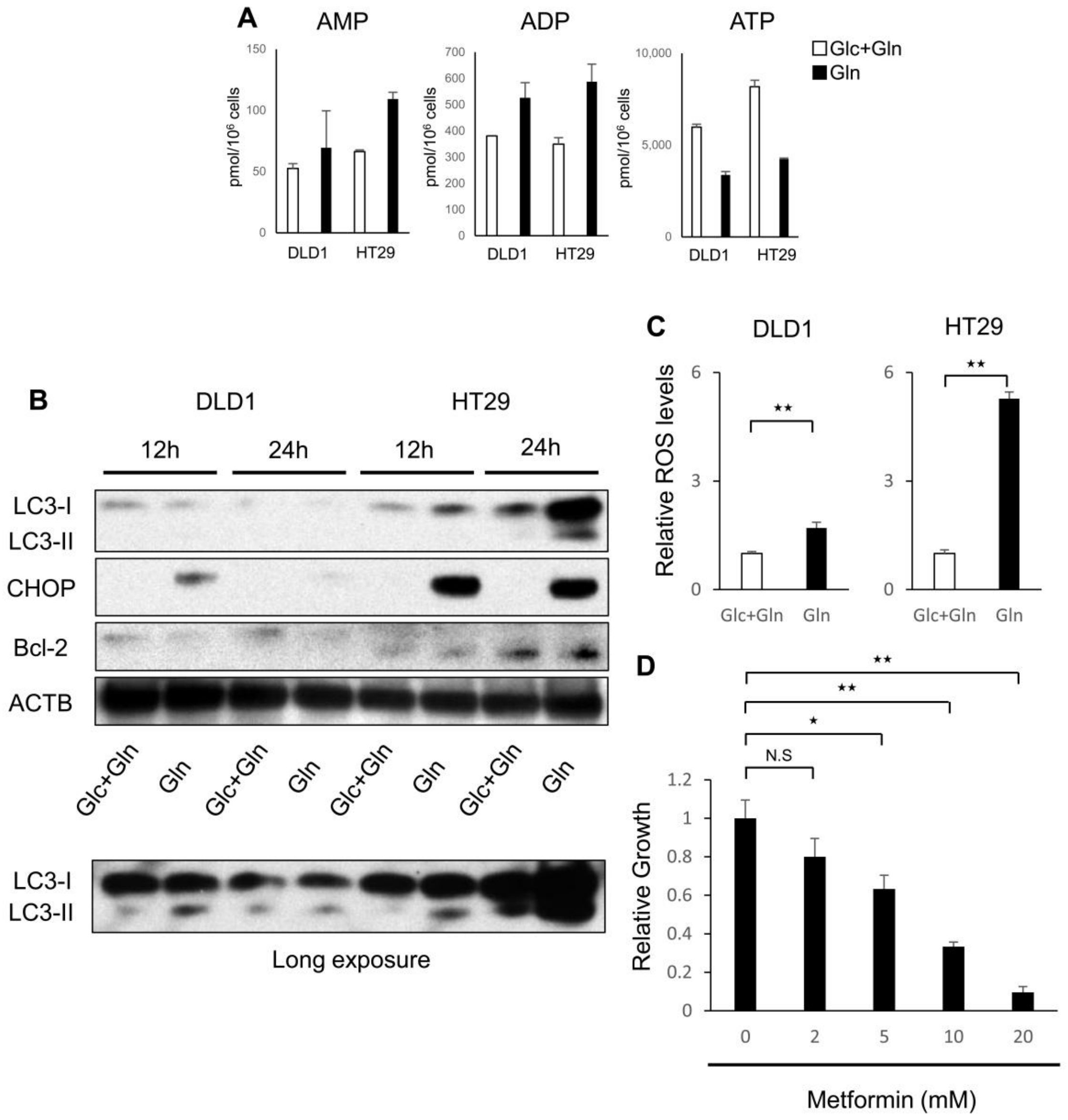

Supplementary Figure S7.

A

Gene symbol	DLD1 fold change	HT29 fold change	Fold change ratio
OGDHL	0.97682	0.34743	2.811556
SDHA	0.719286	0.404749	1.777116
GLUD1	1.237236	0.700324	1.766662
GLUD2	1.045947	0.731532	1.429805
MDH1	1.092697	0.801061	1.364062
SDHC	0.948091	0.713862	1.328115
IDH3B	0.815563	0.639968	1.27438
SDHB	1.04909	0.872917	1.20182
ACO1	0.619135	0.54752	1.130798
IDH2	0.70794	0.642079	1.102574
GOT2	0.633156	0.575184	1.100788
FH	0.57725	0.52854	1.092159
SDHD	0.738022	0.677916	1.088662
DLST	1.031277	1.108913	0.929989
MDH2	0.803871	0.888772	0.904474
ACO2	0.975688	1.104961	0.883007
IDH1	1.319204	1.703627	0.77435
OGDH	0.842993	1.125433	0.749039
IDH3G	0.785161	1.09581	0.716513
IDH3A	0.618151	0.89195	0.693033

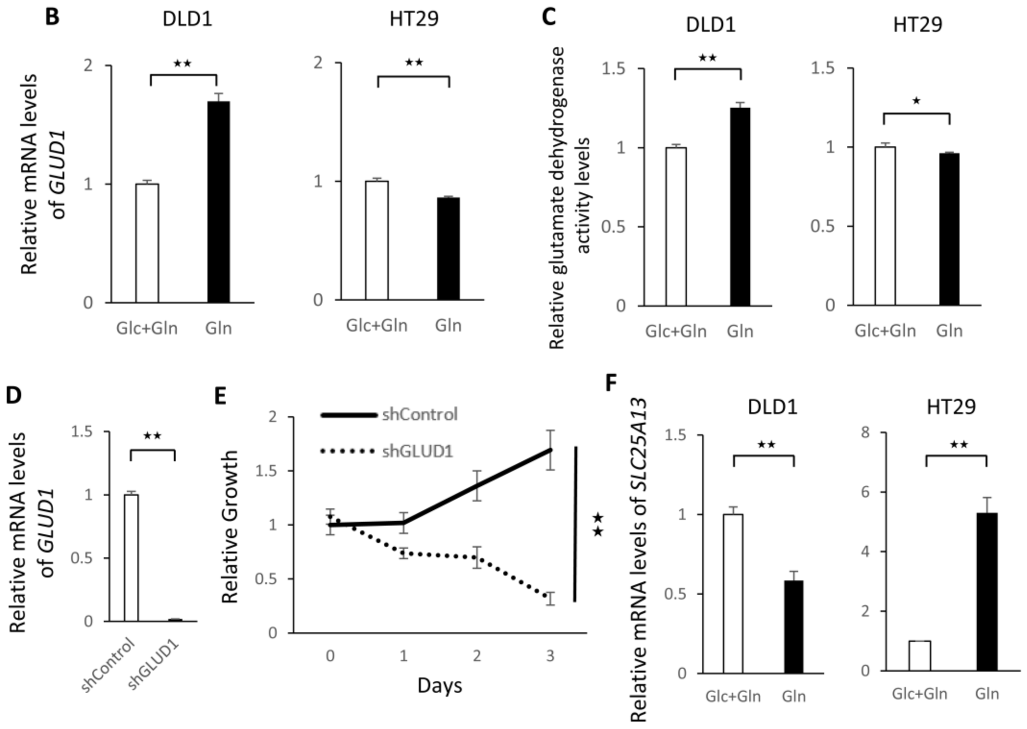

Supplementary Figure S8.

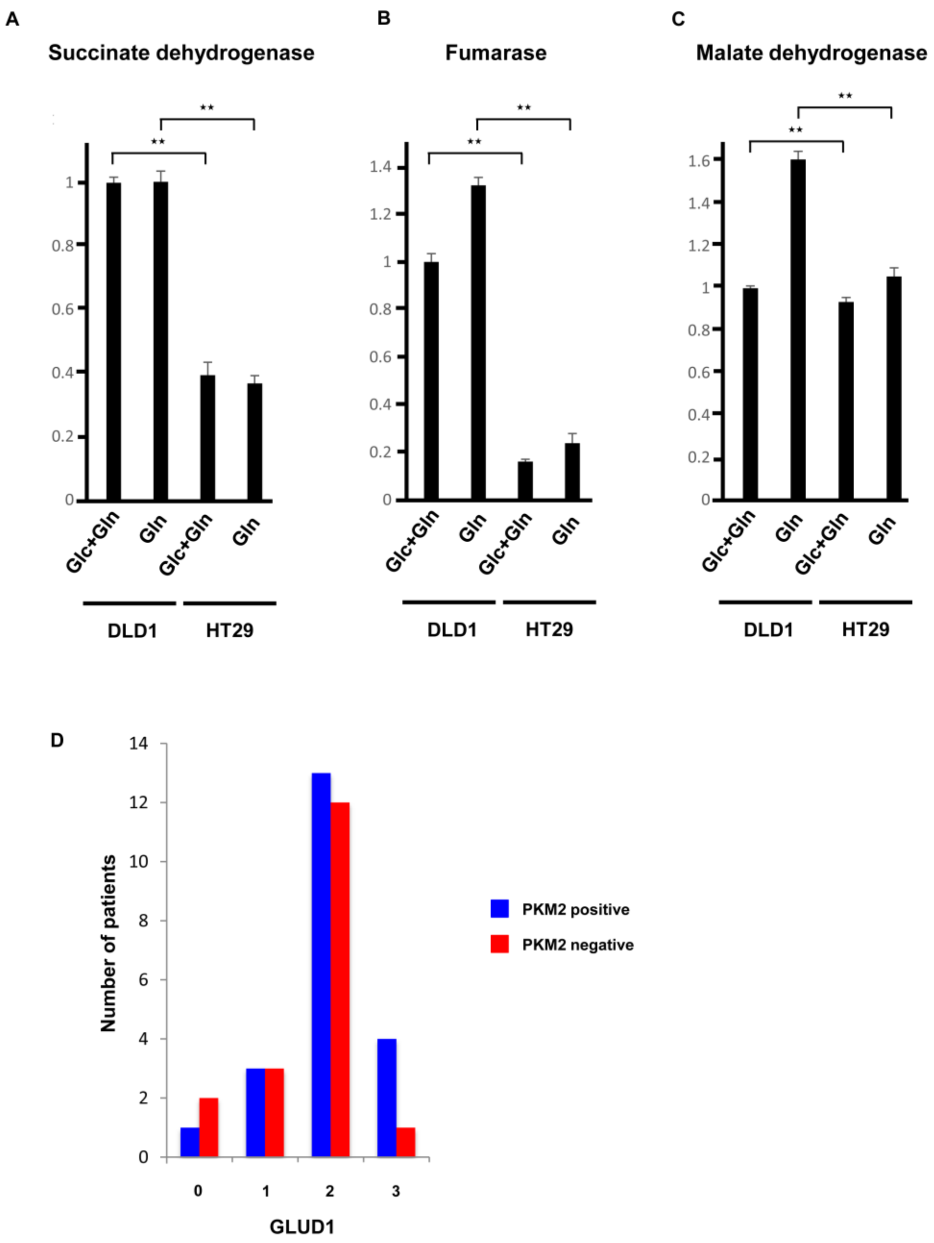

Supplement: Supplementary Information [file srep38415-s1.pdf]
